# Predicting Postpartum Hemorrhage Risk With Second-Trimester Data

**DOI:** 10.1097/og9.0000000000000091

**Published:** 2025-06-12

**Authors:** Mark A. Clapp, Siguo Li, Kaitlyn E. James, Sarah N. Bernstein, Anjali J. Kaimal, Roy H. Perlis

**Affiliations:** Department of Obstetrics and Gynecology and the Department of Psychiatry, Massachusetts General Hospital, and Harvard Medical School, Boston, Massachusetts; and the Department of Obstetrics and Gynecology, University of South Florida, Tampa, Florida.

## Abstract

**OBJECTIVE:**

To assess whether a postpartum hemorrhage (PPH) risk stratification tool using structured data in the electronic health record (EHR) could be developed and validated with information known before the third trimester.

**METHODS:**

We performed a retrospective cohort study of individuals receiving prenatal care and delivering at a single academic institution between January 1, 2017, and December 31, 2023. The cohort included individuals who established care before 24 weeks of gestation, delivered at or after 24 weeks, and had a recorded estimated blood loss at the time of delivery. The cohort was subdivided into three groups for model development: 1) training (66.0% of deliveries between 2017 and 2022), 2) testing (33.0% of deliveries between 2017 and 2022), and 3) temporal validation (deliveries in 2023). We performed traditional and machine-learning modeling approaches in the training data. Discrimination and calibration were compared by calculating the area under the receiver operating characteristic curve and constructing calibration curves; a single approach was selected to identify a predicted probability to classify patients as *high risk of PPH*, which we defined as two or more times the increased risk of PPH and without exceeding a screen-positive rate of 20% (or twice the estimated baseline rate of PPH in the population).

**RESULTS:**

A total of 17,201 patients were included and split between the three cohorts: 10,060 in the training set, 5,183 in the testing set, and 1,958 in the temporal validation set. Model discrimination and calibration were similar among all approaches. Model parsimony was used to select the final modeling approach: logistic regression with stepwise backward selection. This approach yielded an area under the curve of 0.689 (95% CI, 0.667–0.715) and 0.659 (95% CI, 0.621–0.694) in the testing and validation data. Among those classified as high risk, the screen-positive rates and positive predictive values were 19.2% (95% CI, 18.1–20.3%) and 20.1% (95% CI, 17.8–22.7%) in the testing set and 21.6% (95% CI, 19.9–23.4%) and 21.3% (95% CI, 17.7–25.5%) in the validation sets.

**CONCLUSION:**

Using structured EHR data known by 24 weeks of gestation, we developed a prenatal risk stratification model for PPH. This model was designed to be used for predelivery planning and health optimization, particularly among those at the highest risk of PPH and its subsequent morbidity, in contrast to other tools that stratify risk after admission of for delivery.

## Abstract 2

Structured data included in the electronic health record before the third trimester can identify a cohort of people at high risk of postpartum hemorrhage.

Postpartum hemorrhage (PPH) is the most common complication during labor and delivery, estimated to affect up to 3–10% of patients.^1^ It can result in severe maternal morbidity and is attributed to approximately 10% of maternal deaths in the United States.^2,3^ Effective preparation for and management of PPH can mitigate the risk of morbidity.^2,4–6^ Thus, PPH risk stratification during labor and delivery is recognized as an important component of obstetric quality and safety.^7^ Tools for PPH risk stratification such as those developed by Association of Women's Health, Obstetric, and Neonatal Nurses and the American College of Obstetricians and Gynecologists (ACOG), are now commonplace in U.S. labor and delivery units and are designed primarily for risk factor recognition and to facilitate team and unit preparedness.^8,9^

Most risk prediction models for PPH focus on estimating risk during the delivery admission.^10–14^ Few tools are designed for PPH risk stratification before admission for delivery. Predelivery risk stratification may help facilitate health optimization, consultation, or consideration for referral to a center with a higher level of maternal care. Thus, our objective was to determine whether a PPH risk stratification tool using structured data in the electronic health record (EHR) could be developed and internally validated with information known before the third trimester. We hypothesized that many risk factors for PPH are known by the third trimester and that a prediction model could identify a group of individuals at the highest risk of PPH.

## METHODS

We performed a retrospective cohort study of individuals receiving prenatal care and delivering at a single academic institution between January 1, 2017, and December 31, 2023. Uniquely, nearly all patients who deliver at the study institution (aside from transfers) receive prenatal care by a single academic faculty practice comprising midwives, obstetricians, and maternal–fetal medicine specialists; all use a common EHR system (Epic). The cohort included individuals who *established care* (defined as observing a prenatal visit and a complete blood count, a laboratory test ordered universally for all patients initiating care) before 24 weeks of gestation, delivered at or after 24 weeks, and had a recorded estimated blood loss at the time of delivery. If individuals had more than one delivery during the time frame, only one—selected at random—was included. The cohort was subdivided randomly into three groups for model development: 1) training (66.0% of deliveries that occurred between 2017 and 2022), 2) testing (33.0% of deliveries that occurred between 2017 and 2022), and 3) temporal validation (all deliveries in 2023).

The primary outcome of interest was *PPH*, defined as estimated blood loss exceeding 1,000 mL.^15^ All estimated blood losses were reported by clinical staff and recorded in the EHR at the end of the delivery; quantitative blood loss was not used at the study institution during the study period. A list of potential predictor variables that would have been known by 24 weeks of gestation and captured in structured data fields in the EHR was derived after a review of the literature and PPH hemorrhage risk assessment tools (eg, Association of Women's Health, Obstetric, and Neonatal Nurses PPH Risk Tool). The list included maternal age, last recorded weight, last recorded height, last recorded body mass index (BMI, calculated as weight in kilograms divided by height in meters squared), last measured hemoglobin, last measured mean corpuscular volume, last recorded platelet count, observed ferritin value, oral iron listed on the medication list, anticoagulation listed on the medication list, aspirin listed on the medication list, placenta previa or low-lying placenta, multiple gestation, leiomyomas, placenta accreta, prior cesarean delivery, conception through in vitro fertilization, coagulopathy, hemoglobinopathy, nutritional anemia, chronic hypertension, asthma, history of pregnancy complication, history of neonate with *large-for-gestational-age birth weight* (defined as more than 4,000 g), and number of prior vaginal deliveries. The definitions for these items are included in Appendix 1, available online at http://links.lww.com/AOG/E164. Dates for information added and removed to the EHR were used to recreate only the information that was present in the EHR and known on the date corresponding to 23 6/7 weeks of gestation for each patient. Biometric and laboratory data used the most recent value available. Missing data for BMI, the only variable with missing data, were imputed with the population median in the training set. Both variable construction choices were made to facilitate future implementation.

We compared traditional and machine-learning modeling approaches in the training data to determine which approach would balance both model performance and model parsimony, the concept of selecting the simplest model without compromising performance: 1) logistic regression with all predictor variables, 2) logistic regression with backward variable selection (selection parameter: minimization of Akaike Information Criterion), 3) lasso, 4) random forest, and 5) extreme gradient boosting. Discrimination and calibration were compared by calculating the area under the receiver operating characteristic (ROC) curve and constructing calibration curves in the test data set, respectively. The final model was selected by a comprehensive assessment of discrimination, calibration, and parsimony; the performance of this model was evaluated in the temporal validation set.

We a priori set the criteria for success of the final prediction model to identify a group of individuals at two or more times the increased risk of PPH without exceeding a screen-positive rate of 20% (or twice the estimated baseline rate of PPH in the population). Using the training data, we determined the predicted probability associated with the top quintile of predicted risk (ie, the threshold corresponding to a screen-positive rate of 20%). This predicted probability threshold value was applied to the testing and temporal validation set to determine the positive predictive value (PPV), negative predictive value, sensitivity, and specificity.

There are known disparities in maternal morbidity and PPH by race and ethnicity.^16–20^ Thus, we examined whether the model demonstrated similar performance within these subgroups. Using the probability threshold derived from the overall cohort, we reported the screen-positive rate and PPV by race and ethnicity in the test data set. For subgroups without comparable results to the overall population, we compared the prevalence of risk factors in those subgroups with that in the other populations to understand potential drivers of model performance. This study was reviewed and approved by the Mass General Brigham IRB. Modeling was performed with R and Stata software. Values of *P*<.05 were considered statistically significant.

## RESULTS

There were 20,522 patients who had at least one delivery between January 1, 2017, and December 1, 2023, of which 468 (0.2%) had no estimated blood loss recorded and were excluded. Of the remaining 20,054 patients, 2,853 (14.2%) had no laboratory data before 24 weeks of gestation (ie, patients transferring care into the practice or with no prenatal care in the first or second trimester) and were excluded. A total of 17,201 patients were included and split between the three cohorts: 10,060 in the training set, 5,813 in the testing set, and 1,958 in the temporal validation set.

Characteristics among the training, testing, and validation sets are shown in Table 1. There were no significant differences in maternal or pregnancy characteristics between the training and testing sets (*P*>.05), as expected because of the random allocation. However, the external temporal validation set did differ in certain aspects from the training data. Patients in the validation set were slightly younger (median±SD age 32.6±4.9 years vs 33.0±4.9 years, *P*=.01), were more likely of Hispanic ethnicity (22.1% vs 19.0%, *P*=.004), had higher BMI (mean 29.1±7.1 vs 27.8±5.7, *P*<.001), and were more commonly prescribed oral iron (21.7% vs 17.2%, *P*<.01) and aspirin (8.7% vs 5.3%, *P*<.001). There were more nulliparous patients (85.3% vs 74.6%, *P*<.001) and fewer with a history of a prior cesarean delivery (10.7% vs 14.4%, *P*<.001). All variables that were significantly different between the training and validation sets are noted in Table 1.

**Table 1.** Comparison of Patient Characteristics for the Training, Testing, and Validation Data Sets*

Variable	Data Set			*P*	
	Training (n=10,060)	Testing (n=5,183)	Validation (n=1,958)	Testing vs Training	Training vs Validation
Demographics					
Age (y)	33.0±5.0	33.0±4.9	32.6±4.9	.94	.01
Race†				.35	.25
American Indian/Alaska Native	5 (0.05)	6 (0.12)	2 (0.1)		
Asian	1,226 (12.2)	629 (12.1)	223 (11.4)		
Black	720 (7.16)	385 (7.4)	163 (8.3)		
Multiracial or additional races	1,571 (15.6)	771 (14.9)	328 (16.8)		
White	6,318 (62.8)	3,260 (62.9)	1,199 (61.2)		
Unknown	220 (2.2)	132 (2.5)	43 (2.2)		
Ethnicity†				.95	.004
Hispanic	1,912 (19.0)	987 (19.0)	433 (22.1)		
Non-Hispanic	7,770 (77.2)	3,996 (77.1)	1,462 (74.7)		
Unknown	378 (3.8)	200 (3.9)	63 (3.2)		
Biometric data					
BMI (kg/m^2^)	27.8±5.7	28.0±6.2	29.1±7.1	.34	<.001
Missing	1,897	989	813		
Laboratory data					
Hemoglobin (g/dL)	12.44±1.02	12.46±1.02	12.35±1.08	.10	<.001
Mean corpuscular volume (g/dL)	88.95±5.3	88.92±5.2	88.43 (5.3	.70	<.001
Platelet count (10^3^/mL)	258.6±58.5	257.9±56.8	267.3 (58.2	.48	<.001
Observed ferritin laboratory test	572 (5.7)	288 (5.6)	170 (8.7)	.77	<.001
Medication list data					
Oral iron	1,726 (17.2)	858 (16.6)	425 (21.7)	.36	<.001
Anticoagulation	210 (2.1)	101 (1.9)	40 (2.0)	.61	.97
Aspirin	537 (5.3)	306 (5.9)	171 (8.7)	.16	<.001
Pregnancy history					
Neonate with LGA birth weight	385 (3.8)	190 (3.7)	51 (2.6)	.65	.01
Prior cesarean delivery	1,450 (14.4)	734 (14.2)	209 (10.7)	.69	<.001
Prior vaginal deliveries				.17	<.001
0	7,505 (74.6)	3,777 (72.9)	1,671 (85.3)		
1	1,785 (17.7)	965 (18.6)	188 (9.6)		
2	535 (5.3)	314 (6.1)	64 (3.3)		
3	171 (1.7)	93 (1.8)	22 (1.1)		
4 or more	64 (0.6)	34 (0.7)	13 (0.7)		
Pregnancy complication‡	1,017 (10.1)	546 (10.5)	197 (10.1)	.43	<.001
Pregnancy or medical conditions					
Accreta	102 (1.0)	59 (1.1)	44 (2.2)	.53	<.001
Asthma	1,545 (15.4)	818 (15.8)	307 (15.7)	.51	.74
Chronic hypertension	510 (5.1)	260 (5.0)	104 (5.3)	.92	.70
Coagulopathy	335 (3.3)	165 (3.2)	57 (2.9)	.66	.38
Leiomyomas	456 (4.5)	242 (4.7)	106 (5.4)	.73	.10
Hemoglobinopathy	433 (4.3)	226 (4.4)	81 (4.1)	.91	.78
In vitro fertilization	335 (3.3)	147 (2.8)	143 (7.3)	.11	<.001
Multiple gestation	232 (2.3)	121 (2.3)	40 (2.0)	.96	.53
Nutritional anemia	262 (2.6)	114 (2.1)	70 (3.6)	.14	.02
Previa or low-lying placenta	1,038 (10.3)	545 (10.5)	154 (7.9)	.77	.001

BMI, body mass index; LGA, large for gestational age.

Data are mean±SD or n (%) unless otherwise specified.

* The training and testing data sets were randomly derived from the same underlying data set. Variables were compared between the two training and testing data sets, which were all *P*>.05, and between the training and validation data sets.

† Race and ethnicity are shown to aid in the interpretation of the generalizability of this study, but they were not used as predictor variables in the prediction models.

‡ History of pregnancy complications was identified by the International Classification of Diseases, Tenth Revision code Z87.59, which is a fairly generic diagnosis code but does include conditions such as history of fetal death.

**Fig. 1.**
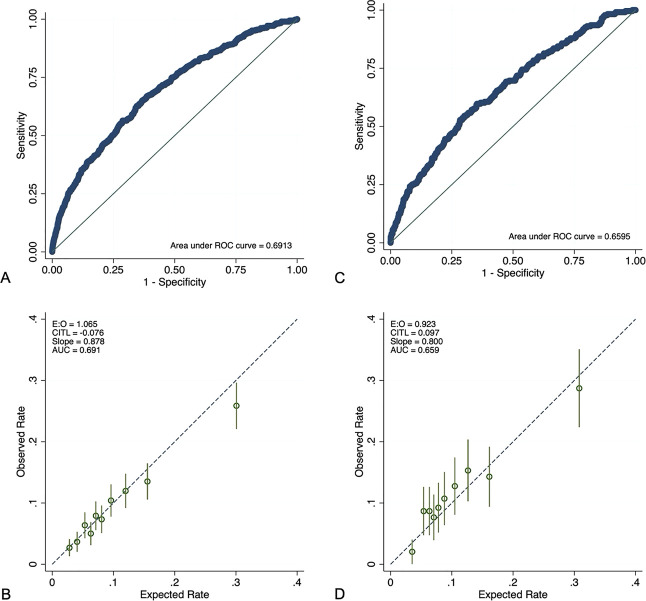
Receiver operating characteristic (ROC) curves and calibration plots between the testing (**A** and **B**) and validation (**C** and **D**) data sets. E:O, expected:observed; CITL, calibration-in-the-large; AUC, area under the curve.

The areas under the curve for the different modeling approaches are shown in Table 2. Discrimination was similar for all models in the testing data, ranging from 0.689 to 0.692, and all models exhibited comparable calibration. The ROC curves and calibration plots for all approaches are shown in the Appendices 2–6, available online at http://links.lww.com/AOG/E164. Given the similarities in their performance, model parsimony was used to select the final modeling approach: logistic regression with stepwise backward selection. This model contained 14 predictors. The model equation and coefficients are included in Appendix 7, available online at http://links.lww.com/AOG/E164. This approach yielded an area under the curve of 0.689 (95% CI, 0.667–0.715) in the testing data and 0.659 (95% CI, 0.621–0.694) in the validation data and demonstrated an ability to stratify those at the highest risk of PPH. The ROC curve and calibration plot for testing and validation data for this approach are shown in Figure 1.

**Table 2.** Comparison of Discrimination Between the Modeling Approaches and Among the Three Data Sets

Model	No. of Predictors	Area Under the Receiver Operating Characteristic Curve (95% CI)		
		Training Set	Testing Set	Validation Set
Logistic regression	23	0.711 (0.694–0.728)	0.692 (0.667–0.714)	0.658 (0.616–0.697)
Lasso	22	0.711 (0.693–0.728)	0.692 (0.667–0.718)	0.659 (0.620–0.696)
Backward selection*	14	0.711 (0.693–0.727)	0.691 (0.667–0.715)	0.659 (0.621–0.694)
Random forest	23	0.724 (0.707–0.740)	0.689 (0.665–0.714)	0.644 (0.604–0.681)
Extreme gradient boosting	23	0.761 (0.746–0.775)	0.689 (0.664–0.712)	0.663 (0.624–0.699)

* The logistic regression model, in which the terms were chosen through backward selection, was selected as the final model used in the analysis.

*Clapp. Predicting PPH Risk With Second-Trimester Data. Obstet Gynecol 2025*.

The factors and their unadjusted and adjusted odds ratios (aORs) in the final model are shown in Table 3. The factors with the highest odds of PPH included multiple gestation (aOR 6.09, 95% CI, 4.58–8.09), accreta (aOR 2.41, 95% CI, 1.43–3.91), placenta previa (aOR 1.81, 95% CI, 1.50–2.19), and prior cesarean delivery (aOR 1.77, 95% CI, 1.50–2.09). A history of vaginal deliveries was associated with lower odds of PPH compared with no history of vaginal delivery.

**Table 3.** Unadjusted and Adjusted Odds Ratios for Postpartum Hemorrhage From the Backward Stepwise Logistic Regression Model

Variable*	OR (95% CI)	aOR (95% CI)
Maternal age (y)†	1.07 (1.05–1.08)	1.05 (1.03–1.06)
BMI†	1.06 (1.04–1.07)	1.05 (1.03–1.06)
Hemoglobin (g/dL)†	0.93 (0.87–0.98)	0.92 (0.86–0.98)
Supplemental oral iron on medication list‡	1.21 (1.03–1.42)	1.20 (1.01–1.42)
History of neonate with LGA birth weight‡	1.57 (1.16–2.08)	1.48 (1.06–2.04)
Leiomyomas‡	2.24 (1.75–2.83)	1.73 (1.33–2.24)
Accreta‡	2.67 (1.65–4.17)	2.41 (1.43–3.91)
Prior cesarean delivery‡	2.54 (2.19–2.95)	1.77 (1.50–2.09)
In vitro fertilization‡	2.50 (1.89–3.25)	1.76 (1.31–2.33)
Hemoglobinopathy‡	1.47 (1.11–1.93)	1.26 (0.92–1.69)
Chronic hypertension‡	2.16 (1.71–2.71)	1.51 (1.17–1.94)
Prior vaginal deliveries^,^§		
0	Ref	Ref
1	0.42 (0.33–0.51)	0.41 (0.32–0.51)
2	0.46 (0.31–0.65)	0.41 (0.27–0.59)
3	0.46 (0.23–0.83)	0.37 (0.17–0.70)
4 or more	0.84 (0.35–1.73)	0.48 (0.18–1.08)
Previa or low-lying placenta‡	1.77 (1.47–2.11)	1.81 (1.50–2.19)
Multiple gestation‡	6.92 (5.27–9.06)	6.09 (4.58–8.09)

OR, odds ratio; aOR, adjusted odds ratio; BMI, body mass index; LGA, large for gestational age; Ref, reference.

* Variable definitions are included in Appendix 1, available online at http://links.lww.com/AOG/E164.

† Continuous variable.

‡ Binary variable.

§ Categorical variable.

Using the final model, we selected the predictive probability that correlated to a screen-positive rate of 20% (chosen a priori to reflect twice the estimated baseline risk of PPH in the overall population) from the training set. Individuals with a predicted probability greater than this threshold were labeled as at high risk of PPH. This threshold was applied to the testing and validation sets to determine whether the screen positive was comparable and to evaluate the sensitivity, specificity, PPV, and negative predictive value for the high-risk designation. The screen-positive rates and PPV were 19.2% (95% CI, 18.1–20.3%) and 20.1% (95% CI, 17.8–22.7%) in the testing set and 21.6% (95% CI, 19.9–23.4%) and 21.3% (95% CI, 17.7–25.5%) in the validation set. Other performance characteristics are shown in Appendix 8, available online at http://links.lww.com/AOG/E164.

Table 4 shows the prevalence of PPH, the screen-positive rate, and the PPV using the high-risk designation in the test group by race and ethnicity. Individuals who self-identified as Black had a significantly higher prevalence of PPH than individuals of other races (15.1% vs 9.0%, *P*<.001). In general, the screen-positive rate and PPV were similar among races and non-Hispanic and Hispanic ethnicities except among individuals who self-identified as Black; among this group, 29.4% were classified as high risk, and the PPV for PPH was 29.2%, which was two times the baseline rate in the population.

**Table 4.** Comparison of Prevalence of Postpartum Hemorrhage and Screen-Positive Rates and Positive Predictive Values for the High-Risk Group in the Test Data Set

Group	No. of Individuals	Prevalence	Screen-Positive Rate	PPV
Overall	5,183	9.4 (9.7–10.3)	19.2 (18.2–20.3)	20.1 (17.7–22.7)
Subgroups				
Race*				
Asian	629	7.8 (5.9–10.1)	15.6 (12.9–18.6)	15.3 (9.3–24.0)
Black	385	15.1 (11.8–19.0)	29.4 (25.0–34.1)	29.2 (21.5–38.3)
Multiracial or additional races	771	8.5 (6.8–10.8)	16.5 (14.0–29.2)	19.7 (13.6–27.6)
White	3,260	9.4 (8.4–10.4)	19.5 (18.2–20.9)	19.4 (16.5–22.6)
Unknown	132	9.1 (5.2–15.4)	17.4 (11.8–24.9)	17.4 (6.3–39.7)
Ethnicity*				
Hispanic	987	8.5 (6.9–10.4)	16.3 (14.1–18.8)	19.2 (13.8–26.1)
Non-Hispanic	3,996	9.0 (8.9–9.1)	19.7 (18.5–20.6)	20.2 (17.5–23.2)
Unknown	220	7.5 (4.8–12.1)	24.0 (18.6–30.4)	20.8 (11.6–34.6)

PPV, positive predictive value.

Data are % (95% CI) unless otherwise specified.

* Race and ethnicity data in the electronic health record were obtained from self-report. American Indian/Alaska Native subgroup is not shown because of the small denominator (n=6).

To understand potential contributors to the performance of the model in this subgroup, we compared the prevalence of the risk factors in the final model in the training data among Black and non-Black patients. There were notable differences in the hemoglobin (mean 11.8 g/dL vs 12.5 g/dL, *P*<.001), leiomyomas (12.1% vs 4.0%, *P*<.001), prior cesarean delivery (21.8% vs 13.8%, *P*<.001), and hemoglobinopathies (20.0% vs 3.1%, *P*<.001) in Black compared with non-Black patients, respectively. Other differences are shown in Appendix 9, available online at http://links.lww.com/AOG/E164.

## DISCUSSION

A common obstetric complication, PPH affects approximately 10% of deliveries at the study institution, a large academic referral center. Using structured data captured in the EHR and known before 24 weeks of gestation, we built and temporally validated a prediction model for PPH. Although the overall model had modest discrimination, it achieved our preset performance characteristic goal (ie, PPV more than 20% at a screen-positive threshold of 20%) when the predictive probabilities were dichotomized into a high-risk and a low-risk group. In our final model, the screen-positive rates and PPV were 19.2% and 20.1% in the testing set and 21.6% and 21.3% in the temporal validation sets, respectively. We did identify that our high-risk designation disproportionately identified a higher proportion of Black patients as high risk compared with other race groups. This was attributed to a difference in the prevalence of many risk factors used in the predicted model; however, given that this group also has approximately 1.6 times increased risk of PPH in the study population (approximately 15% vs 9%), we believe the increased percentage of Black patients labeled as at high risk of PPH appropriately reflects their underlying increased risk of PPH, and no model adjustments were made post hoc.

In this study, we designed a PPH risk stratification approach for the third trimester. This tool contrasts with many existing PPH risk prediction tools such as the Association of Women's Health, Obstetric, and Neonatal Nurses and ACOG tools, which are focused on PPH risk in the intrapartum period and assist care teams in identifying risk factors that are present or evolve over a patient's labor and delivery.^8,9^ Although these tools are important for labor and delivery units, they are not specifically designed to be used before admission; thus, their applicability is unknown. Risk stratification in the third trimester could be used to aid in predelivery health optimization, to prompt consultations or referrals, and to allow preadmission unit preparation (eg, to ensure appropriate staffing overage or blood product availability). One intended use of this tool is to guide targeted anemia and iron deficiency screening in the third trimester for individuals at high risk of PPH because many patients present with anemia at delivery despite the current recommended approach to screening (ie, third-trimester complete blood count).^21,22^ We hypothesize this may be an effective approach to iron deficiency screening (ie, screening a group of patients at high risk of PPH to prevent or correct iron-deficiency anemia) as opposed to universal screening, which currently lacks data or support from the U.S. Preventive Services Task Force.^22^

To facilitate prospective validation and implementation, we deliberately designed and constructed a model using data that are easily extractable in the EHR. Therefore, we limited predictors to information found in structured data fields such as diagnoses in the medical history or problem list or medications added to the patient's medication list. Although this approach does not capture free-text data in clinical documentation, it is more adaptable to EHR systems and less reliant on clinician documentation styles or practices, which may vary by clinician or obstetric clinic.^23^ Although this was a retrospective study, another strength of this modeling approach was the recreation of only the EHR information entered and known before 24 weeks of gestation, thereby more realistically simulating a prospective risk prediction. Similarly, variables such as mode of delivery (cesarean or vaginal) or estimated fetal size, which may increase the risk of PPH but are not necessarily known in the first or second trimester, were not included as candidate predictor variables to increase the translatability of this model to practice.^1,24^

We made the intentional choice to define PPH as an estimated blood loss exceeding 1,000 mL per ACOG clinical guidelines.^15^ This outcome definition has similarly been used in other models and risk prediction tools for PPH.^8–11,25^ We also set this outcome definition according to our intended use of the model prediction, which will ultimately be used in a study that prospectively screens individuals at high risk of PPH for iron deficiency and anemia throughout the third trimester. We recognize that the risk of morbidity from PPH is correlated with the amount of blood loss (ie, higher blood loss results in higher risk of morbidity) and that individuals or institutions may find varying definitions for PPH or clinically significant PPH (eg, PPH requiring transfusion or estimated blood loss exceeding 1,500 or 2,000 mL) more useful, depending on their intended use for the prediction. The same modeling approaches described here could be used for other definitions of PPH, which is facilitated with structured data inputs in a widely used EHR system.

Because we designed this model for implementation in our practice, data used to build, test, and temporally validate this model were derived from a single institution. Although this ensures that the model is well constructed for application within the study institution, it limits the generalizability of these exact model specifications and variable weights to generate predictive probabilities at other institutions; external validation studies are needed. Although some of the strengths and limitations of structured data were previously acknowledged, this reliance does require the information to be added to the EHR (ie, updating the medical record structured data fields to reflect a patient's current and accurate health state). This study used data from patients who had established prenatal care within a single faculty practice that used a common EHR, thereby reducing the risk of, but not excluding, unreliable or incorrect information in the EHR. The use of recorded estimated blood loss in the delivery record limits the applicability of this prediction to immediate PPH; delayed-onset PPHs were not reliably captured. The study expanded multiple years, in which covariates were changing over time; although we demonstrated temporal validity, the model may need to be recalibrated over time if there are continued shifts in the underlying population characteristics to ensure that its performance is maximized.

Using structured EHR data known by 24 weeks of gestation, we developed a prenatal risk stratification model for PPH. In the temporal validation data set, 21.6% were classified as at high risk of PPH; the PPV for PPH for this high-risk classification was 21.3%. This model was designed to be used for predelivery planning and health optimization, particularly among those at the highest risk of PPH and its subsequent morbidity, in contrast to other tools that stratify risk after admission. Although intentionally designed for integration into the EHR, further prospective and external validation studies are needed to verify model performance before its widespread adoption outside the study institution.
